# Combinatorial Therapy of Cancer: Possible Advantages of Involving Modulators of Ionic Mechanisms

**DOI:** 10.3390/cancers14112703

**Published:** 2022-05-30

**Authors:** Mustafa B. A. Djamgoz

**Affiliations:** 1 Department of Life Sciences, Imperial College London, South Kensington Campus, London SW7 2AZ, UK; m.djamgoz@imperial.ac.uk; Tel.: +44-796-181-6959; 2 Biotechnology Research Centre, Cyprus International University, Haspolat, Mersin 10, Turkey

**Keywords:** ion channel, exchanger, surgery, chemotherapy, radiotherapy, targeted therapy, immunotherapy

## Abstract

**Simple Summary:**

Cancer, which is a major health problem, is a complex disease. Currently, the main treatment methods are surgery, chemotherapy, radiotherapy and biological therapies. The latter include hormonal therapies, inhibitors of growth-promoting tyrosine kinase enzymes, and immunotherapy which aims to activate the immune system to destroy tumors. Whilst all these methods work, efficacy is often limited in time (with tumors gradually becoming resistant to treatment). Furthermore, undesirable side effects, which can seriously curtail quality of life, are common. Consequently, in addition to new treatment modalities constantly being developed, it is even more expedient to make existing therapies more effective by combining them with each other or with other agents. Here, we evaluate the evidence for the effectiveness of combining conventional cancer treatments with modulators of ionic mechanisms, mainly channels that permeate sodium, calcium and potassium. We conclude, in every case, that such combinations can produce improved outcome by making given treatments more effective and reducing the undesirable side effects. In addition, ionic modulators by themselves can exert anti-cancer effects.

**Abstract:**

Cancer is a global health problem that 1 in 2–3 people can expect to experience during their lifetime. Several different modalities exist for cancer management, but all of these suffer from significant shortcomings in both diagnosis and therapy. Apart from developing completely new therapies, a viable way forward is to improve the efficacy of the existing modalities. One way is to combine these with each other or with other complementary approaches. An emerging latter approach is derived from ionic mechanisms, mainly ion channels and exchangers. We evaluate the evidence for this systematically for the main treatment methods: surgery, chemotherapy, radiotherapy and targeted therapies (including monoclonal antibodies, steroid hormones, tyrosine kinase inhibitors and immunotherapy). In surgery, the possible systemic use of local anesthetics to suppress subsequent relapse is still being discussed. For all the other methods, there is significant positive evidence for several cancers and a range of modulators of ionic mechanisms. This applies also to some of the undesirable side effects of the treatments. In chemotherapy, for example, there is evidence for co-treatment with modulators of the potassium channel (Kv11.1), pH regulation (sodium–hydrogen exchanger) and Na^+^-K^+^-ATPase (digoxin). Voltage-gated sodium channels, shown previously to promote metastasis, appear to be particularly useful for co-targeting with inhibitors of tyrosine kinases, especially epidermal growth factor. It is concluded that combining current orthodox treatment modalities with modulators of ionic mechanisms can produce beneficial effects including (i) making the treatment more effective, e.g., by lowering doses; (ii) avoiding the onset of resistance to therapy; (iii) reducing undesirable side effects. However, in many cases, prospective clinical trials are needed to put the findings firmly into clinical context.

## 1. Introduction

Although cancer is a major global health problem, with 1 in 2–3 people expected to be diagnosed with some form of it during their lifetime, many problems remain in its clinical management. These problems include limitations in definitive functional diagnoses, seen most clearly in using prostate ‘specific’ antigen (PSA) for detecting prostate cancer. Mammography, used commonly also for screening purposes, can misdiagnose breast cancer, often in the direction of overdiagnosis [1,2]. Even the latest major treatment modality and the most promising immunotherapy has limitations [3]. Consequently, there is serious need to improve existing clinical treatment modalities.

One possibility, already in routine practice, is to combine existing cancer drugs. These are then encoded often by the first letters of the drugs in the combination. For example, CHOP (cyclophosphamide, doxorubicin, vincristine and prednisone) for non-Hodgkin’s lymphoma; CMF (cytoxan, methotrexate and fluorouracil) for breast cancer; BEP (bleomycin, etoposide and cisplatin) for testicular cancer.

A novel type of cancer mechanism to emerge more recently and proving to be of clinical relevance is related to ion transporting proteins (ITPs). The latter are comprised mainly of ion channels (voltage-sensitive, ligand-gated or mechanosensitive), exchangers and pumps. Indeed, a wide range of ITPs are expressed in cancer cells and tissues and contribute dynamically to the different stages of the cancer process, from initial proliferative activity to invasion and metastasis (e.g., [4,5]). Such major ITP mechanisms include K^+^ channels controlling proliferation, voltage-gated sodium channels (VGSCs) that promote invasiveness and metastasis and pH, which has wide-ranging actions. It is well known that cancer cells have an alkaline cytoplasm and develop pericellular acidity in order to invade surrounding tissues (e.g., [6]). A major mechanism controlling tumor pH is sodium–hydrogen exchanger-1 (NHE1) (e.g., [7]). ITPs offer significant advantages as clinical anti-cancer targets due to their well understood functional attributes and pharmacology, derived in part, from many years of progress in neuroscience. In fact, a new term has emerged recently—*cancer neuroscience* [8,9]. This concept manifests itself in several ways including expression of neuronal antigens, voltage-gated ion channels, membrane excitability/generation of action potentials and direct innervation of tumors [9]. All these are generating novel treatment possibilities, often non-invasive and with minimal side effects. In the case of ion channels, there is already available a huge battery of agents (blockers and openers) that can be employed against various stages and mechanisms of the cancer process. In fact, some ~15% of currently used drugs target ITPs and these can readily be ‘repurposed’ as cancer drugs, bringing into the clinic their significant advantages of well-known pharmacokinetics and safety. Any such success would be a major boost to patients, especially those with high risk of metastatic disease.

**Figure 1:**
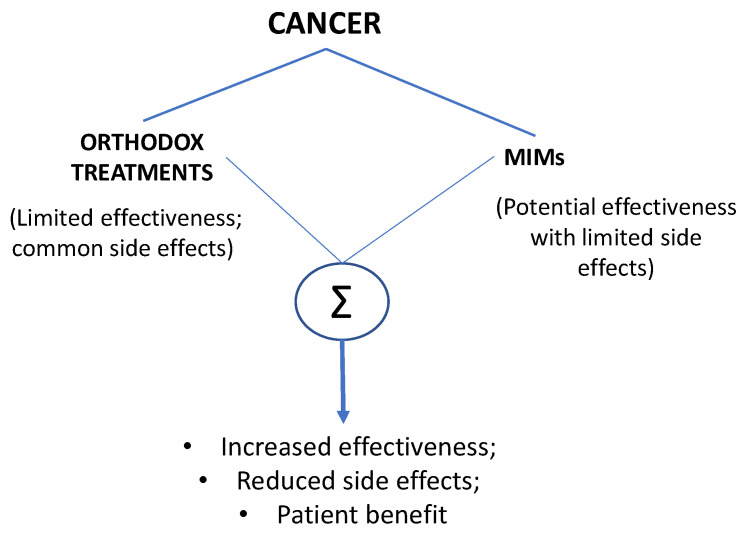
A conceptual representation of the relative advantages of combining orthodox treatments of cancer with modulators of ionic mechanisms (MIMs). ‘Σ’ indicates combination.

In this perspective, we aim to evaluate the case for combining clinical cancer drugs with agents acting on ITPs, i.e., modulators of ionic mechanisms (MIMs). This is with a view to generating synergy and overcoming some of the problems associated with orthodox treatments of cancer, altogether for improved patient outcomes (Figure 1). Our aim is not to generalize the application of MIMs to all types of cancer since the latter represents a group of diseases with diverse etiology, progression, possible treatment and fate. Consequently, MIMs could act differently on different cancers and combine diversely with different treatments. As a consequence, different results with the same blocker might be detectable in different tumors. Thus, our aim is not to provide an exhaustive summary of this field. Instead, we review the progress made mainly in the last decade, highlighting some of the best understood cases.

## 2. Therapeutic Modalities

In this section, we deal with the major clinical modalities of cancer treatment one by one. Then, we review and discuss how each could be combined with MIMs, as defined in the Introduction. The aim is to evaluate possible bases for generating synergy.

### 2.1. Surgery

Surgical removal of cancer when small and detected early may be the best way of eliminating the disease. In clinical cancer surgery, however, there is a phenomenon called ‘showering’ whereby some cancer cells escape and spread during the excision. Such rogue cells may seed somewhere and can proliferate after some time, especially if the micro-environment is immunosuppressed. The use of local anesthesia would seem a logical opponent of this process since (i) local anesthetics (LAs), such as lidocaine, are well known blockers of VGSCs and (ii) there is significant evidence that VGSC activity inhibits cancer cell invasiveness and metastasis [10].

An early meta-analysis did suggest that regional anesthesia could improve overall survival after oncologic surgery [11]. Clinical aspects of this topic were analyzed subsequently in further detail in a series of articles published in 2021 and 2022 (https://www.frontiersin.org/research-topics/21757/anesthesia-and-cancer-friend-or-foe#articles—accessed on 15 April 2022). The pre-clinical case seemed strong, with LAs suppressing the viability and migratory capacity of breast cancer cells, dependent on the concentration and duration of drug application [12,13,14,15]. At least a part of the suppressive effect of LAs could occur via VGSC inhibition [16]. Disappointingly, however, regional anesthesia with lidocaine during tumor resection did not appear to have a significant effect on breast cancer outcomes including recurrence [17]. Nevertheless, it remained plausible that other anesthetic techniques (e.g., total intravenous anesthesia and systemic LA infusion) might influence the oncologic outcome in the resection surgery of other major tumors (e.g., colorectal and lung) [17]. Indeed, a recent meta-analysis of cohort studies concluded that the use of a combined epidural–general anesthesia during surgery on patients with colon cancer could improve overall survival [18]. It may also be possible to apply a ‘local anesthetic’ intravenously to patients already receiving general anesthesia for surgery. A retrospective review of patients undergoing primary debulking surgery for ovarian cancer concluded, albeit with some limitations, that intraoperative intravenous lidocaine infusion was associated with improved disease-free survival and overall survival [18]. Similarly, intraoperative intravenous lidocaine infusion during radical cystectomy for bladder cancer appeared to be associated with prolonged overall and disease-free survival [19]. These promising results should be interpreted cautiously and need to be confirmed by subsequent randomized clinical trials. Interestingly, VGSCs may not be the only type of ion channel related to oncological surgery. Retrospective univariate and multivariate analyses showed that hERG1 expression had a significant negative association with disease recurrence in surgically resected clear cell renal carcinoma [20].

In overall conclusion, the current data do generate hypotheses and promising evidence about the influence of MIMs, including anesthetic agents, on cancer progression. Importantly, however, further prospective trials that determinate causality are necessary before changing the current clinical practice becomes a reality [21,22,23]. In this regard, it is promising to see an ongoing randomized controlled trial to assess the impact of VGSC blockade during surgery on disease-free survival, to report in 2026 [https://clinicaltrials.gov/ct2/show/NCT01916317—accessed on 15 April 2022].

### 2.2. Chemotherapy

There are two broad classes of chemotherapeutic drugs, both targeting fast-dividing cells. *Anthracyclines* kill cancer cells by DNA intercalation and damage. Cell death can follow through several mechanisms. These include the formation of free radicals (e.g., reactive oxygen species; ROS) leading to oxidative stress. *Taxanes* interfere with cancer cells’ ability to divide by disruption of the cellular mitotic machinery (e.g., microtubules). Although chemotherapy aims to target cancer cells, significant damage to proliferative normal cells also often occurs. Accordingly, a major incentive in ‘combinatorial’ chemotherapy is to boost the killing of cancer cells whilst reducing the undesirable side effects and trying to avoid the build-up of drug resistance [24]. In these respects, combinatorial treatment involving coupling with ionic mechanisms, especially ion channels, offers significant potential [25,26].

A wide range of ion channels can influence chemotherapy but in different ways in different cancers. For example, there was a positive correlation between Kv11.1 (hERG) expression and chemosensitivity of breast, lung and gastric cancer (mainly in vitro) to vincristine, camptothecin, paclitaxel and cisplatin [27,28]. On the other hand, for acute lymphoblastic leukemia (cell lines, primary cell culture and in vivo mouse), the correlation with Kv11.1 expression was negative for doxorubicin, prednisone and methotrexate (Pillozzi et al., 2011). The case for colon cancer seemed more mixed [27,29]. Other ion channels, cancers and chemotherapeutic drugs are discussed in detail by Altamura et al. [25].

Acidity (pH) can also affect the effectiveness of chemotherapy. Indeed, several chemotherapeutic agents might work better under alkaline conditions. These include aphidicolin, vinblastine sulfate, paclitaxel, aclarubicin and trichostatin A [30,31]. Accordingly, such treatments would benefit from combination with NHE1 inhibitors. The latter have been shown to improve the temozolomide chemotherapy of glioma [32]. In another complementary experiment, Amith et al. [33] generated a stable NHE1-knockout of the highly invasive, triple-negative, MDA-MB-231 breast cancer cells. In vitro, the modified cells had markedly lower rates of migration and invasion; in vivo, xenograft tumor growth was significantly decreased. Silencing of functional NHE1 expression also increased the susceptibility of the cells to paclitaxel-mediated cell death as well as further decreased the viability and migratory and invasive capabilities of the cells. Such effects were not seen in hormone (estrogen) sensitive, weakly/non-invasive MCF-7 cells. These data suggested that NHE1 inhibitors could serve as novel partners in chemotherapy for triple-negative breast cancer, one of the most difficult to treat subtypes of this cancer.

Another relevant ionic mechanism is the Na^+^-K^+^ pump (ATPase), which is blocked by cardiac glycosides [34]. Several cardiac glycosides were tested on different colorectal cancer cell lines and primary tumor cells from patients alone and in combination with cytotoxic drugs (5-fluorouracil, oxaliplatin, cisplatin, irinotecan) [35]. In particular, the combination of digitoxin, which is used in cardiac disease, and oxaliplatin exhibited synergism including against the highly drug-resistant HT29 cell line. However, it was not clear if the concentration of digitoxin used (IC50 > 0.27 μM ≡ 211 ng/mL) could be achieved in patient plasma since the clinical dose is normally ca. 117 ng/mL [36].

Various MIMs can also suppress the build-up of resistance to chemotherapy [37,38]. Underlying these are a range of ITPs including transient receptor potential (TRP), K^+^ and Cl^−^ channels [38]. As a promoter of chemoresistance, the best evidence is for the Orai/Stim complex of Ca^2+^ signaling [39].

**Figure 2:**
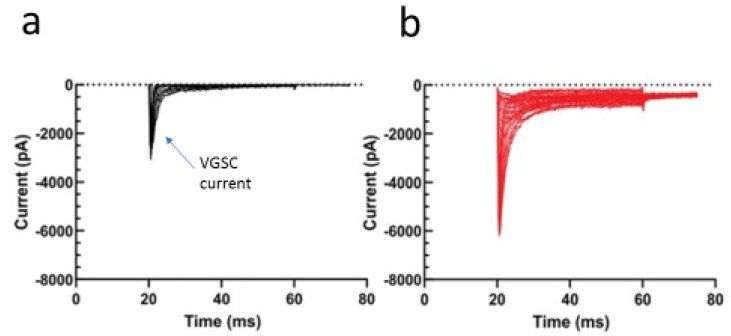
Enhancement of peak and late currents produced by endogenous voltage-gated sodium channels in rat DRG neurons by paclitaxel (PTX). Endogenous Nav1.7 currents were elicited by 40 ms depolarizing voltage steps from −80 mV to +10 mV in 5 mV increments from a holding potential of −100 mV. (**a**) Control data from neurons treated with DMSO. (**b**) Data from neurons treated with 25 nM PTX. Modified from Akin et al. [45].

Importantly, as well as synergizing with the effects of chemotherapy, MIMs can also reduce its side effects. Thus, Minoti [40] demonstrated clinically that the undesirable side effects of anthracyclines and nonanthracycline chemotherapeutics on heart function can be reduced by using ranolazine, an inhibitor of the VGSC persistent current promoted by hypoxia [41]. As regards nerves, oxaliplatin was shown initially to suppress VGSC activity in neurons [42,43]. More recently, treatment of rats with paclitaxel in vivo was shown to induce long-term *upregulation* of Nav1.7 mRNA and protein expression in dorsal root ganglia [44]. A comparable result was obtained from humans [14]. Importantly, there was a corresponding increase in channel activity (Figure 2) [45]. Such chemotherapy-induced VGSC upregulation could be one of the consequences of peripheral nerve damage resulting in neuropathic pain. Accordingly, co-treatment with VGSC inhibitor drugs (e.g., lacosamide) could reduce a major side effect of chemotherapy [26,46]. Another drug of interest is minoxidil, which was shown to be neuroprotective against paclitaxel-induced peripheral neuropathy in a mouse model [47]. Minoxidil is an opener of ATP-gated/closed (K_ATP_) K^+^ channels and could also inhibit VGSC activity by hyperpolarizing the membrane potential.

The impact of MIMs on chemotherapy may also manifests itself in cancer patients taking antihypertensive medications that include certain Ca^2+^ channel blockers (CCBs). This topic was recently reviewed [48,49]. Results were mixed but overall suggested that the use of CCBs alone had no effect on cancer progression and outcome. Their co-application with chemotherapy, however, revealed some synergistic effects. For example, co-treatment with CCBs (nifedipine or amlodipine) potentiated gemcitabine chemotherapy in pancreatic cancer in vitro and in vivo [49]. As drug repurposing becomes more viable, this area of investigation could generate further significant leads that could benefit patients.

In overall conclusion, MIMs offer treble potential in cancer management. First, agents such as VGSC blockers can directly inhibit cancer progression [10]. Indeed, ranolazine has been proposed as an anti-metastatic drug [50]. Second, MIMs can improve the effectiveness of chemotherapy, possibly due, in part, to their direct action on cancer cells, mentioned above. Third, co-treatment with MIMs can be beneficial to patients also by suppressing peripheral neuropathy, one of most debilitating side effects of chemotherapy.

### 2.3. Radiotherapy

Brain tumors are commonly treated by radiation and glioblastoma express a variety of channels. These include aquaporins (AQPs 1, 4 and 9), voltage-gated K^+^, Na^+^ and Ca^2+^ channels, Cl^−^ channels, acid-sensing ion channels and TRP channels [51]. There is evidence for the involvement of a range of ion channels and MIMs in response to radiation, especially as regards induced tumor cell death [52]. Ionic mechanisms also play a significant role in resistance to radiotherapy [53].

In particular, ionizing radiation in doses used clinically for fractionated radiotherapy has been shown to activate K^+^ channels, which, in turn, can contribute to the DNA damage response and promote survival of the irradiated tumor cells [54,55]. The blockade of K_Ca_ (BK) channels inhibited radiation-induced migration of glioblastoma multiforme (GBM) cells into surrounding regions of the brain in an orthotopic mouse model [56]. Klumpp et al. [57] showed for GBM in vitro that the K_Ca_3.1 channel was involved in resistance to radiotherapy, mesenchymal glioblastoma stem cells being the responsible subpopulation of cells. The inhibition of K_Ca_3.1 channel with TRAM-34 also sensitized GBM to temozolomide treatment in a syngeneic mouse glioma model [58]. He et al. [59] focused on the voltage-gated K^+^ channel subfamily, KCNQ1OT1, which is upregulated in several cancers. The depletion of KCNQ1OT1 could significantly re-sensitize lung adenocarcinoma cells to stereotactic body radiotherapy by inhibiting autophagy, dependent on miR-372-3p. A comparable study on liver cancer revealed a similar scenario in vivo but involving miR-146a-5p [60]. On the other hand, one microM Imipramine (a tricyclic antidepressant and blocker of Kv10.1) did not affect the radiosensitivity of DU145 prostate cancer cells [61]. Skonieczna et al. [62] showed that the voltage-dependent anion channel (VDAC) controls the cellular response to ionizing radiation through modulation of the ROS- and nitric oxide-dependent signaling pathways in irradiated K562 lymphoblastoid cells. The inhibition of VDAC with 500 µM 2′-disulfonic acid (DIDS) induced apoptosis to enhance the effectiveness of radiotherapy. A similar radiosensitization effect was observed for mitochondrial VDACs in human myelogenous leukemia cells [63].

Ion channels also manifest themselves in other aspects of radiotherapy, for example, as follows:

i. *Radiodermatitis*. This is a painful side effect of radiotherapy where irradiation of the skin causes inflammation and breakdown of the epidermis and can lead to significant morbidity. There is some evidence for the role of ion channels in this process (e.g., [64]). This appears to involve decreased levels of systemic inflammatory cytokines, e.g., IL-1β and IL-6 [59].

ii. *Chemoradiation*. Cancers are frequently treated with a combination of chemotherapy and radiotherapy, sometimes after surgery. In these cases, also, MIMs can play a significant role (e.g., [65]). This is probably due to a combination of MIM effects seen in chemotherapy and radiotherapy.

iii. *Abscopal effect*. This is the phenomenon where localized radiotherapy (even sub-therapeutic dosages) can also cause significant immunostimulatory regression of distant, non-irradiated tumors. There is some evidence that MIMs could play a significant role in this process (e.g., [66]). Gap junctional transmission, known to be controlled by Ca^2+^ and H^+^, could drive this effect. [67]

iv. *Ligand-gated channels*. The ionic fluxes permeated by such receptors can also contribute to the efficacy of radiotherapy. For example, expression of the purinergic P2X7 receptor, associated with cationic fluxes, can even predict GBM radiosensitivity and survival [68].

v. *Normal cells*. Some work has also been conducted on non-tumor cells. Thus, Xia et al. [69] showed that glibenclamide (a K_ATP_ channel blocker) prevented radiation-induced lung injury and inhibited radiation-induced apoptosis of vascular endothelial cells by increased Ca^2+^ influx and the subsequent activation of PKC.

### 2.4. Targeted Therapies

These comprise a broad range and aim to suppress the cancer process by impacting specific mechanistic targets. Targeted therapies have been approved by the FDA for more than 15 types of cancer, including those of breast, prostate, colon and lung. There are two main types of targeted therapies: monoclonal antibodies (mAbs) and small-molecule drugs (SMDs). In turn, the latter can be tyrosine kinase inhibitors or modulators of hormone action. 

#### 2.4.1. Monoclonal Antibodies

Probably the best-known anti-cancer mAb is trastuzumab (Herceptin) which is used against breast cancer positive for HER2, a member of the epidermal growth factor receptor (EGFR) family. This is evaluated here as a test case. Trastuzumab has been combined with MIMs in relation to both improving drug efficacy and reducing side effects, especially on heart function. Some emphasis has been placed upon the Ca^2+^-activated Cl^−^ channel, CaCC (TMEM16A/ANO1). Fujimoto et al. [70] studied the impact of CaCC activity on HER2 transcription in the HER2-resistant breast cancer cell line, YMB-1. Measured by the WST-1 method, the viability of the control cells, which were HER2-positive, was not affected by trastuzumab. Pharmacological and siRNA-mediated inhibition of ANO1 significantly prevented HER2 transcription in YMB-1 cells. These results suggest (i) that ANO1 may function as a transcriptional regulator of HER2 and (ii) that ANO1 inhibitors have potential in the treatment of breast cancer patients with resistance to HER2-targeted therapy.

HER2 is also expressed in other solid tumors [71]. Kulkarni et al. [72] studied the role of CaCC in head and neck squamous cell carcinoma (HNSCC) in vitro and in tumor xenografts. The application of cetuximab (CTX, an EGFR-targeting mAb) and simultaneous suppression of CaCC led to a pronounced loss of cell viability. In further experiments CaCC was pharmacologically inhibited or a non-conducting form of the channel was expressed [65]. The results suggested that the channel regulated EGFR and HER2 signaling in the cellular pathways controlling growth and survival. Accordingly, in the absence of functional CaCC, tumor cells may acquire resistance to clinical inhibitors and activating CaCC improves the response to biological therapies targeting members of the EGFR/HER family [72].

#### 2.4.2. Steroid Hormones

Several steroid hormones (SHs) play a critical role in a range of cancers. The best-known cases are estrogen and testosterone for breast and prostate cancers, respectively. The impact of SHs on ITPs generally were recently reviewed extensively by Restrepo-Angulo et al. [73]. Overall, many ITPs are under the control of SHs at a hierarchy of levels from transcription to post-translation. We should note that whilst SH action is classically genomic and slow-acting, fast effects also occur via membrane-bound receptors [74]. The latter topic is outside the scope of the current account. The relevance of the SH-ITP connection to the cancer process was reviewed earlier by Fraser et al. in relation to VGSC involvement [75].

The main question to consider in this section is whether MIMs can associate with SH-based treatments. Again, our aim is not to give an exhaustive account of this topic. Instead, we highlight some key findings and issues relating to breast and prostate cancers, the two most common hormone-sensitive cancers.

A particularly interesting case concerns VGSCs that promote and may even initiate metastasis [10]. Some key findings to date relating to breast cancer are as follows:

i. The estrogen receptor antagonist, tamoxifen, inhibited VGSC activity in the SHG-44 glioma cell line and in rat cortical neurons with an IC50 of 5.54 micromol/L [76,77].

ii. Similar effects of ‘selective estrogen receptor modulators’ (SERMs), including raloxifene (‘Evista’), were seen on rodent hypothalamic neurons and ventricular myocytes [78,79,80].

iii. VGSC protein has a binding site for tamoxifen [81]. This site mediates the inhibitory effect of tamoxifen and its analogues via stabilization of the non-conducting inactivated state of the channel.

Interestingly, the weakly metastatic MCF-7 cells express estrogen receptors (ERs) but no functional VGSC; in contrast, the strongly metastatic MDA-MB-231 cells lack ER but express functional VGSCs, which promote invasive behavior [82]. This suggests that estrogen signaling may downregulate functional VGSC expression. This notion was supported by the results of transfection experiments. Thus, transfecting ERα into MDA-MB-231 cells downregulated VGSC (neonatal Nav1.5) mRNA expression [75]. Conversely, silencing ER in MCF-7 cells revealed an electrophysiologically detectable and pharmacologically distinguishable VGSC current [83].

Prostate and breast cancers share many similarities [84]. Essentially, what has been said for breast cancer can also be extended to prostate cancer. Thus, the pathophysiology of prostate cancer also involves VGSC expression and its pro-metastatic role [85,86]. Functional expression occurs specifically in cells that are hormone resistant [68].

Some interesting clinical possibilities emerge from this generalized evidence. First, since the anti-cancer effects of SH signaling may occur via the inhibition of VGSC activity, it may be possible to enhance the effectiveness of hormone therapy by combining with ‘repurposable’ drugs. Second, it is possible that VGSC expression occurs when breast cancer becomes hormone refractory. If so, it may be possible to treat a hormone-resistant disease with VGSC blockers. This may be extended to ‘combinatorial’ treatments. For example, we showed recently (i) that minoxidil, an opener of K_ATP_ channels, produces anti-invasive effects on MDA-MB-231 cells and (ii) that this synergizes with the effect of the VGSC persistent current blocker ranolazine under hypoxic conditions [87]. Obata [88] showed that tamoxifen similarly induces the opening of K_ATP_ channels. On the one hand, this evidence is broadly supportive of the Celex hypothesis of metastasis, i.e., that it is membrane excitability resulting from the concurrent upregulation of VGSC and downregulation of K^+^ channels that promote invasiveness [50]. On the other hand, it could form the basis of a triple combination: tamoxifen, ranolazine and minoxidil.

In addition to the VGSC connection, Ca^2+^ signaling can contribute to SH resistance in breast cancer. Thus, hormone-independent and -resistant cells were found to overexpress Ca^2+^ channels; in hormone-resistant cells, treatment that combined a CCB with an antiestrogen (tamoxifen) reversed resistance to the antiestrogen [89]. A comparable situation was found in prostate cancer [90]. The involvement of Ca^2+^ signaling in SH resistance may be associated with VGSC activity since these two ionic mechanisms are intimately related, directly through Na^+^-Ca^2+^ exchange or via the membrane voltage [91].

#### 2.4.3. Tyrosine Kinase Inhibitors

Tyrosine kinases are integral parts of growth factor (GF) receptors which are well known to be involved in cancer initiation and progression [92]. Consequently, GF receptors and their associated downstream signaling cascades are major targets for cancer therapy [93]. In humans, receptor tyrosine kinases (RTKs) comprise some 20 subfamilies [92]. Most work has been conducted with epidermal GF (EGF).

In breast cancer cells, the role of EGF in promoting migratory behavior was shown to be mediated substantially through functional VGSC expression, i.e., TTX suppressed the increase in cellular migration induced by EGF dose dependently [94]. In non-small lung cancer (NSCLC), also, Campbell et al. [95] showed that 100 ng/mL EGF induced a significant increase in invasiveness and this was blocked completely by 1 µM TTX or gefinitib (“Iressa”). Thus, it was concluded that the pro-invasive effect of EGF on NSCLC occurred mainly through VGSC / Nav1.7 activity.

In breast cancer BT-474 cells, both astemizole (a Kv10.1/EAG1 K^+^ channel blocker) and gefitinib induced cell cycle arrest and inhibited proliferation dose dependently (IC50 = 1.72 µM and 0.51 µM, respectively). [96] Importantly, co-treatment with the two drugs produced a synergistic effect with a combination index of 0.75 to 0.26. These studies reinforced the notion that combining tyrosine kinase inhibitors (TKIs) with MIMs can significantly improve anti-cancer treatments. This could also help to avoid the build-up of resistance to TKI treatment. Indeed, Choi et al. [97] showed that the sensitivity of drug-resistant human lung adenocarcinoma cell line (NCI-H460) to gefinitib (2 µM) in suppressing cell viability (measured by MTT assay) was improved by co-treatment with general Kv channel blockers (TEA and 4-AP), through inhibition of the Ras-Raf signaling pathway. A similar effect was obtained with treatment with ca. 100 nM dendrotoxin-κ (DTX-κ), a blocker of Kv1.1, in vitro and in a xenograft model [98].

In head and neck squamous cell carcinoma (HNSCC), EGFR was found to be in a mutually interactive protein–protein complex with CaCC (ANO1/TMEM16A) [99]. ANO1 enabled membrane stability of EGFR signaling, which upregulated ANO1 expression in the membrane. HNSCC cell lines with amplification and high-level expression of ANO1 showed enhanced sensitivity to gefitinib, and co-inhibition of EGFR and ANO1 had an additive suppressive effect on cellular proliferation.

Another TKI used commonly against NSCLC and pancreatic cancer is erlotinib (Tarceva). Similar to gefinitib, the effectiveness of erlotinib could be enhanced by coupling with blockers of ion channels including K_Ca_3.1 [100] and ANO9/TMEM16J [101].

From the available evidence, we can conclude the following: (i) GF receptors and ITPs frequently co-occur interactively in cancer cells. (ii) A significant part of GF signaling occurs via ion channels, especially VGSCs. (iii) Anti-cancer effects of TKIs can be potentiated by co-treatment with MIMs. (iv) Expression of the relevant ionic mechanisms can serve as a predictive marker for the response and such a co-treatment strategy might circumvent the development of resistance to single agent therapy [102]. In overall conclusion, there is a strong case for combining MIMs (including ion channel inhibitors) with conventional GF-based therapies. Furthermore, there is similar, albeit less complete, evidence for other GFs, including insulin-like growth factor, nerve growth factor, vascular endothelial growth factor and fibroblast growth factors [75]. These can also associate with a range of MIMs to promote the cancer process through VGSC expression/activity [75]. Finally, one of the problems in tackling cancer with TKIs is the GF ‘redundancy’, i.e., blocking one GF pathway may activate another [103]. In this regard, also, blocking the downstream ‘hub’ (e.g., VGSC) through which signaling may occur could generate an additional advantage [75].

#### 2.4.4. Immunotherapy

Immunotherapy could also be potentiated by combinations with modulators of ionic mechanisms. In a pre-clinical mouse study, Pilon-Thomas et al. applied immunotherapy to pancreatic tumors subcutaneously implanted in mice [104]. Three different immunotherapy modalities were used, anti-CTLA-4, anti-PD1 and adoptive T-cell transfer. One group of mice was fed bicarbonate-enriched water, whilst the control group was given just tap water. Based upon previous work, it was assumed that bicarbonate supplementation would raise intra-tumoral alkalinity [105]. The results showed (i) that bicarbonate alone did not affect survival; (ii) that immunotherapy alone produced survival benefit; (iii) immunotherapy + bicarbonate was significantly more effective than immunotherapy alone in prolonging survival [104]. This phenomenon would be worthwhile investigating in more detail since pancreatic cancer is one of the most difficult cancers to treat. Another such cancer is glioma immunotherapy, which has been shown to be boosted by NHE1 inhibitors [32].

In conclusion, inhibitors of ionic mechanisms controlling (acidifying) the tumor environment, and inducing both local and systemic immunosuppression, would seem ideal candidates for combinatorial immunotherapy. Such a candidate could be the NHE1 inhibitor, cariporide [106].

Interestingly, in breast cancer, and possibly other carcinomas, NHE1 activity is driven by functional voltage-gated sodium channel (VGSC) expression [107]. The latter is well known to promote metastasis in several carcinomas [10]. Accordingly, VGSC blockers are also possible candidates for combination with immunotherapeutic agents. This notion is supported by studies associating checkpoint inhibition with epithelial–mesenchymal transition (EMT), which occurs at the start of the metastatic cascade [108]. Dongre et al. [109] related the efficiency of the anti-CTLA4 immunotherapy of breast cancer to EMT status in an orthotopic tumor model. Thus, tumors in an ‘epithelial’ state were found to be more susceptible than ‘mesenchymal’ tumors. Furthermore, in ‘mixed’ tumors, even a small minority population (10%) of mesenchymal cells could ‘cross-protect’ the majority (90%) of the epithelial component. EMT has also been associated with the activation of other checkpoint molecules, including PD-L1 [110]. Importantly, EMT is controlled in part by VGSC activity [111,112]. Taken together, the available evidence would suggest that checkpoint inhibition coupled with VGSC blockage could also be a viable combinatorial approach to eradicating early-stage solid tumors. Amongst these are ‘repurposable’ drugs, such as ranolazine [50]. The latter is an inhibitor of the VGSC ‘persistent current’, which increases under hypoxic conditions that also promote EMT [113]. In another interesting recent study, by reducing STAT1 activation, inhibition of Na^+^/K^+^-ATPase by cardiac glycosides (ouabain and digoxin) was shown to suppress expression of the immune checkpoint IDO1 in lung and breast cancer cells [114].

## 3. Conclusions and Future Perspective

From the available evidence, we conclude that the currently employed clinical cancer treatment modalities can be made more effective by combination with modulators of ionic mechanisms, thereby potentially improving patient outcomes. Combinations involving MIMs can also suppress some of the undesirable side effects of the treatments. Furthermore, since MIMs can themselves serve as anti-cancer drugs, combinations can even be trebly beneficial. Regarding MIMs, we covered mainly ion channels as the key elements. In fact, from the emerging evidence, the breadth of the appropriate MIMs is likely to be much wider and may even extend to natural products. However, in many instances, the mechanistic insights and subtype(s) of the ion channels (e.g., VGSC) involved are lacking. These need to be elucidated in detail before the full potential of combinatorial therapies of cancer can be attained. Thus, whilst the principle of this particular type of combinatorial therapy is established, much more work remains to be performed. First, most of the evidence is pre-clinical, derived from in vitro experiments, and much more in vivo work needs to be carried out. This should ideally be extended to humans, possibly through ‘real world data’ and, ultimately, clinical trials. Second, it is not clear that the dosages of the combined drugs are at the optimal level, and this needs much more detailed investigation. Third, most experiments to date involved simultaneous treatments. It would be interesting to determine whether outcomes could be improved by offsetting the two combined agents in time, i.e., priming with one and then superimposing the other. Finally, we should emphasize that as more and more cancer drugs emerge, more and more viable combinations will be forthcoming. In fact, systematic identification of biomarker-driven drug combinations to overcome resistance seems possible [115]. Increasingly, therefore, it will be desirable to perform these tasks on given cancers with given treatments and specific MIMs, and probe deeper into the possible mechanisms of interaction.
